# Epidemic time series similarity is related to geographic distance and age structure

**DOI:** 10.1016/j.idm.2022.09.002

**Published:** 2022-10-12

**Authors:** Tad A. Dallas, Grant Foster, Robert L. Richards, Bret D. Elderd

**Affiliations:** a Department of Biological Sciences, University of South Carolina, Columbia, SC, 29208, USA; b Department of Biological Sciences, Louisiana State University, Baton Rouge, LA, 70802, USA

**Keywords:** Epidemic similarity, SARS-CoV2, Age structure, Distance decay

## Abstract

**Objective:**

More similar locations may have similar infectious disease dynamics. There is clear overlap in putative causes for epidemic similarity, such as geographic distance, age structure, and population size. We compare the effects of these potential drivers on epidemic similarity compared to a baseline assumption that differences in the basic reproductive number (*R*_0_) will translate to differences in epidemic trajectories.

**Methods:**

Using COVID-19 case counts from United States counties, we explore the importance of geographic distance, population size differences, and age structure dissimilarity on resulting epidemic similarity.

**Results:**

We find clear effects of geographic space, age structure, population size, and *R*_0_ on epidemic similarity, but notably the effect of age structure was stronger than the baseline assumption that differences in *R*_0_ would be most related to epidemic similarity.

**Conclusions:**

Together, this highlights the role of spatial and demographic processes on SARS-CoV2 epidemics in the United States.

## 1 Introduction

The most recent pandemic of severe acute respiratory syndrome coronavirus 2 (SARS-CoV2) has highlighted the pressing need to understand how epidemics emerge and spread, and how epidemic models may be used for control and mitigation efforts. Models are used to estimate parameters of interest, which are then used to calculate composite properties (e.g., basic reproduction number *R*_0_ (Brauner et al., 2021; Ives & Bozzuto, 2021);) and to simulate epidemics under different mitigation scenarios (e.g. (Baker et al., 2020; Hinch et al., 2021; Sun et al., 2020),). However, these composite pathogen properties are not properties of the pathogen alone, but are conditional on the host population. Differences in susceptibility and contact patterns among individuals is critical to pathogen transmission and epidemic trajectories (Yin et al., 2017). Measures of *R*_0_ – quantifying the approximated number of secondary cases from a single case in a wholly susceptible host population – based on temporal case counts can hint at these differences in individual contact and transmission, but could also suggest differences in pathogen strain diversity and numerous other factors contributing to epidemic dynamics (Corcoran et al., 2020; Ives & Bozzuto, 2021). Understanding the processes that lead to differing epidemic dynamics is a pressing research need, as many of these underlying drivers of estimated *R*_0_ may potentially change over time or with different intervention strategies (Islam et al., 2021).

The SARS-CoV-2 pandemic has created a situation where it may be possible to start to disentangle the role of different factors on resulting epidemic trajectories. For one, county-level data on infectious case counts provide a means to compare how epidemics progressed at the county scale, and to compare epidemic trajectories between counties. At a basic level, this allows for the comparison of epidemic trajectories to differences in *R*_0_, as the larger difference in *R*_0_ would suggest that the epidemics should be quite dissimilar in their trajectories. For one, *R*_0_ may be estimated from the epidemic time series itself, such that epidemics with similar *R*_0_ would naturally have similar dynamics. However, *R*_0_ is a simple composite measure estimated from a time series that may belie the influence of mitigation efforts and fluctuating epidemic dynamics (e.g., COVID-19 case counts appeared in distinct waves, while *R*_0_ estimates do not use all waves (Ives & Bozzuto, 2021)). Apart from similarity in *R*_0_ leading to similar epidemics, differences in epidemic trajectories may be driven simply by geographic space between two epidemics. That is, epidemics should be more similar in nearby counties than in distant counties. This could be driven by several interwoven drivers, which may not be reflected in differences in estimated *R*_0_, including spatial autocorrelation in demographics, climatic effects on transmission, differences in mitigation efforts, or the movement of infectious individuals.

But there is an inherent circularity here, in that estimates of *R*_0_ are based on the epidemic trajectories, such that pairwise differences in *R*_0_ between counties should inherently be related to differences in epidemic trajectories. This creates an interesting baseline for comparison. That is, differences in *R*_0_ should hypothetically relate to differences in epidemic trajectory – barring time-varying *R*_0_ and assuming *R*_0_ can be estimated accurately – simply because *R*_0_ is estimated from a portion of the epidemic time series. Here, we explore how epidemic trajectories are related to differences in *R*_0_, and how other important differences between counties may further influence epidemic trajectories. Specifically, epidemic trajectories may differ as a function of geographic distance between counties, and differences in age structure and population size. We find that there is a clear signal of geographic distance and demographic (population size and age structure) dissimilarity on resulting epidemic trajectory differences for a set of 3139 US counties. We compare the strength of these relationships to the potentially circular relationship between epidemic trajectory differences and differences in *R*_0_, finding that age structure dissimilarity is more strongly related to epidemic trajectory similarity compared to differences in *R*_0_. Together, this suggests an important role for age structure to epidemic emergence and progression, and highlights the importance of considering the spatial landscape of infectious disease.

## 2 Methods

### 2.1 COVID-19 epidemic time series data

Time series case data for SARS-CoV-2 were compiled by the Center for Systems Science and Engineering at Johns Hopkins University (Dong et al., 2020) for a set of 3139 United States counties, with recorded case counts every day for the period between January 22, 2020, and May 9, 2022. These data were then rescaled to cases per 100,000 residents based on county population estimates from the United States Census Bureau from 2019 (Loftin, 2019). County age structure data was also obtained from the US Census Bureau (Loftin, 2019), and standardized to sum to one within a given county. Age structure dissimilarity was estimated as the Euclidean distance between two counties in their age structure distributions. Estimates of *R*_0_ were obtained from (Ives & Bozzuto, 2021), which were estimated from the epidemic time series directly.

### 2.2 Dynamic time warping

**Fig. 1:**
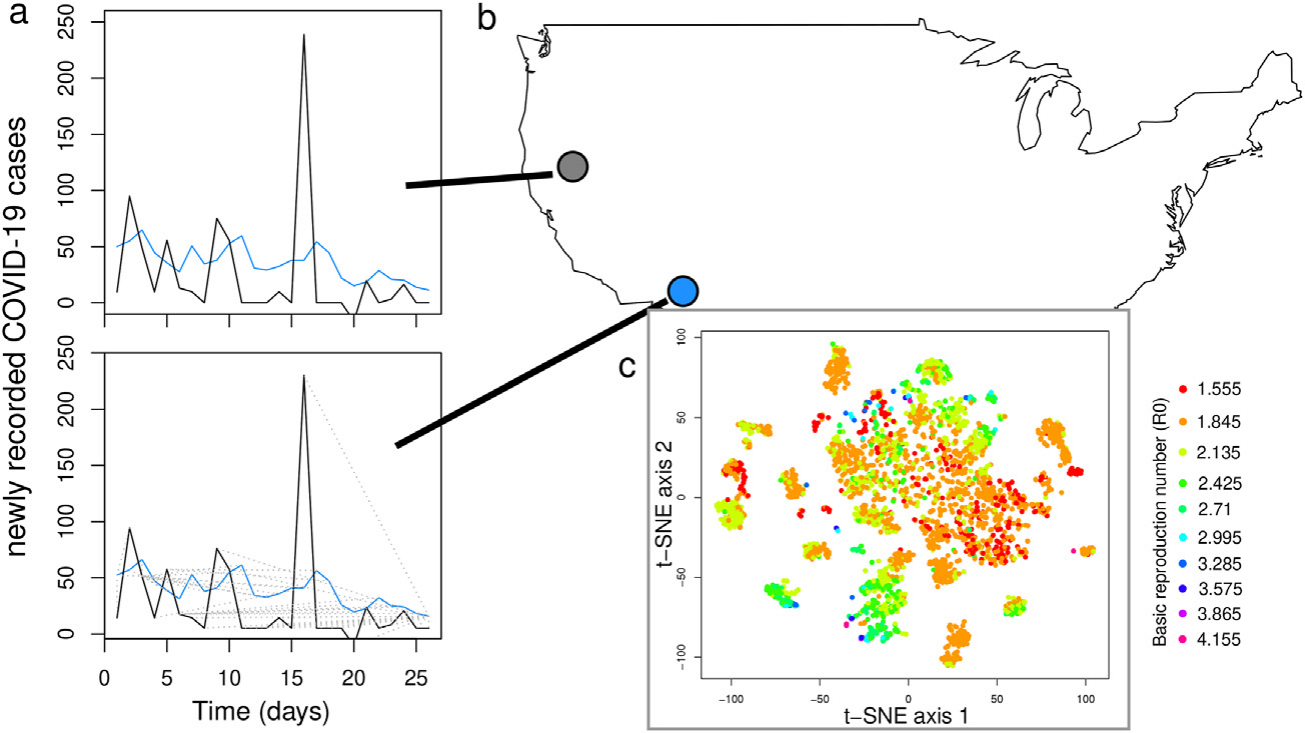
The similarity of epidemic time series was estimated using dynamic time warping, where two time series (in blue and black in panel *a*) are mapped onto one another (indicated by grey lines in panel *a*) to estimate epidemic dissimilarity. Pairwise similarity values were calculated for every pair of counties in the United States (panel *b*). These pairwise values are then compressed to a low-dimensional space by using t-SNE (panel *c*), where each point represents a single US county, and point color corresponds to estimated *R*_0_ from Ives & Bozzuto (2021).

Dynamic time warping (DTW) is an approach to measure the similarity between two time series based on the notion that there is not an inherent 1:1 matching between values in each time series (Berndt & Clifford, 1994), largely applied to problems in speech (Amin & Mahmood, 2008) and gait (Boulgouris et al., 2004) recognition and comparison. The underlying idea is that the speed of speech or gait could be different, while the actual underlying pattern is the same (e.g., the same words can be spoken more quickly or with differing amounts of pauses). In our application to infectious disease, there is no reason to believe that the pairwise difference in COVID-19 case counts between two counties is *actually* a measure of how similar the epidemics are, given that the epidemics may have started at different times. This fundamental disconnect means that perhaps it is more suitable to attempt to match the time series data based on the start of the epidemic or to use an approach which is flexible to different epidemic start times, as we do here. By allowing an *elastic* transformation of the time series, DTW attempts to minimize the difference between the two trajectories while accounting for phase shifts in epidemic dynamics (Fig. 1).

(1) $$DTW\left(x,y\right)=min_{\pi \in A\left(\text{x, y}\right)}{\left(\underset{\left(i,j\right)\in \pi }{\sum }d{\left(x_{i},y_{j}\right)}^{q}\right)}^{1/q}$$

here, we want to compare two epidemic time series (*x* and *y*), considering an alignment path *π* of all possible paths (*A*_*x*,*y*_), where *i* and *j* correspond to the position in the time series mapping onto the potential alignments, where *q* is a normalization constant. The goal is to find an alignment which minimizes the overall dissimilarity between the two time series. We use the dtw R package (Giorgino, 2009), and consider the dissimilarity between the time series to be the normalized cumulative dissimilarity between the two time series. There is a possibility that the results could be sensitive to the inclusion of many leading or trailing zero counts, where epidemics were on a fundamentally different timescale across US counties. While this approach should account for this, we explore the effect of truncating the epidemic time series to include 5 leading and 5 trailing zero values before the calculation of the DTW values. Trimming the time series to remove these zero-values did not affect our findings (see Supplementary Material).

### 2.3 What is related to epidemic similarity?

Epidemic similarity was measured by comparing epidemic time series for every pair of US counties using dynamic time warping as described above. This creates a pairwise dissimilarity matrix, a square matrix estimating epidemic similarity between all US counties. To project this high-dimensional matrix into lower dimensions for analysis, we used t-distributed stochastic neighbor embedding (t-SNE), a method that offers a low-dimensional projection of high-dimensional data (Gisbrecht et al., 2015). The result of this embedding is the production of two t-SNE axes, in which each axis contains one value per US county, and the distance along each axis relates to epidemic dissimilarity, mapping counties out along the two axes. This allows us to relate these low-dimensional axes representing epidemic trajectory similarity to differences between counties in terms of spatial distance, demographics (e.g., age structure and population size), and estimated epidemic properties (*R*_0_ (Ives & Bozzuto, 2021)).

We used Moran's *I* to quantify the effects of geographic distance and age structure dissimilarity on resulting epidemic similarity. That is, how similar are epidemics in different counties as a function of geographic distance between counties or differences in age structure between counties? Originally designed as a measure of spatial autocorrelation, Moran's *I* is essentially a distance-weighted Pearson's correlation, allowing the relationship between a distance matrix (e.g., pairwise geographic distance between all US counties) and a county-level trait (e.g., t-SNE axis values). We related each t-SNE axis – representing the projected epidemic dissimilarity between two US counties – to pairwise matrices of 1) geographic distance between US counties, 2) age structure dissimilarity, 3) absolute difference in population size, and 4) absolute difference in *R*_0_. The underlying idea being that counties that are closer to one another, with similar age structure, and not differing greatly in population size or estimated *R*_0_ (Ives & Bozzuto, 2021) would also be closer together along t-SNE axes. All distance and dissimilarity matrices – describing the relative difference in geographic distance, age structure, population size, and *R*_0_ among US county pairs – were standardized to be bound between 0 and 1, and inverted, such that the largest distances corresponded to the smallest values. This allows us to calculate *z*-scores based on the null distributions, and to compare these scores across the different distance/dissimilarity matrices.

However, we are fundamentally limited by the almost inherent collinearity between some of these measures. For instance, geographic distance and age structure dissimilarity were positively related, based on a Mantel test (*z* = 247, *p* = 0.001), suggesting that more distant counties also have more dissimilar age structure. We explore this further in the Supplemental Materials, where we use Mantel tests on the pairwise epidemic dissimilarity matrix directly, instead of attempting to project the dissimilarity into two axes using t-SNE. However, regressions of distance matrices are notoriously error-prone (Legendre et al., 2015), which is why we present the analyses of the t-SNE axes here. By compressing epidemic similarity into a low-dimensional space, more traditional regression techniques can be used. The results of both analyses are qualitatively similar (see Supplementary Materials for further discussion).

### 2.4 Reproducibility

*R* code and data to reproduce the analyses is provided at

https://doi.org/10.6084/m9.figshare.19782406.v1.

## 3 Results

**Fig. 2:**
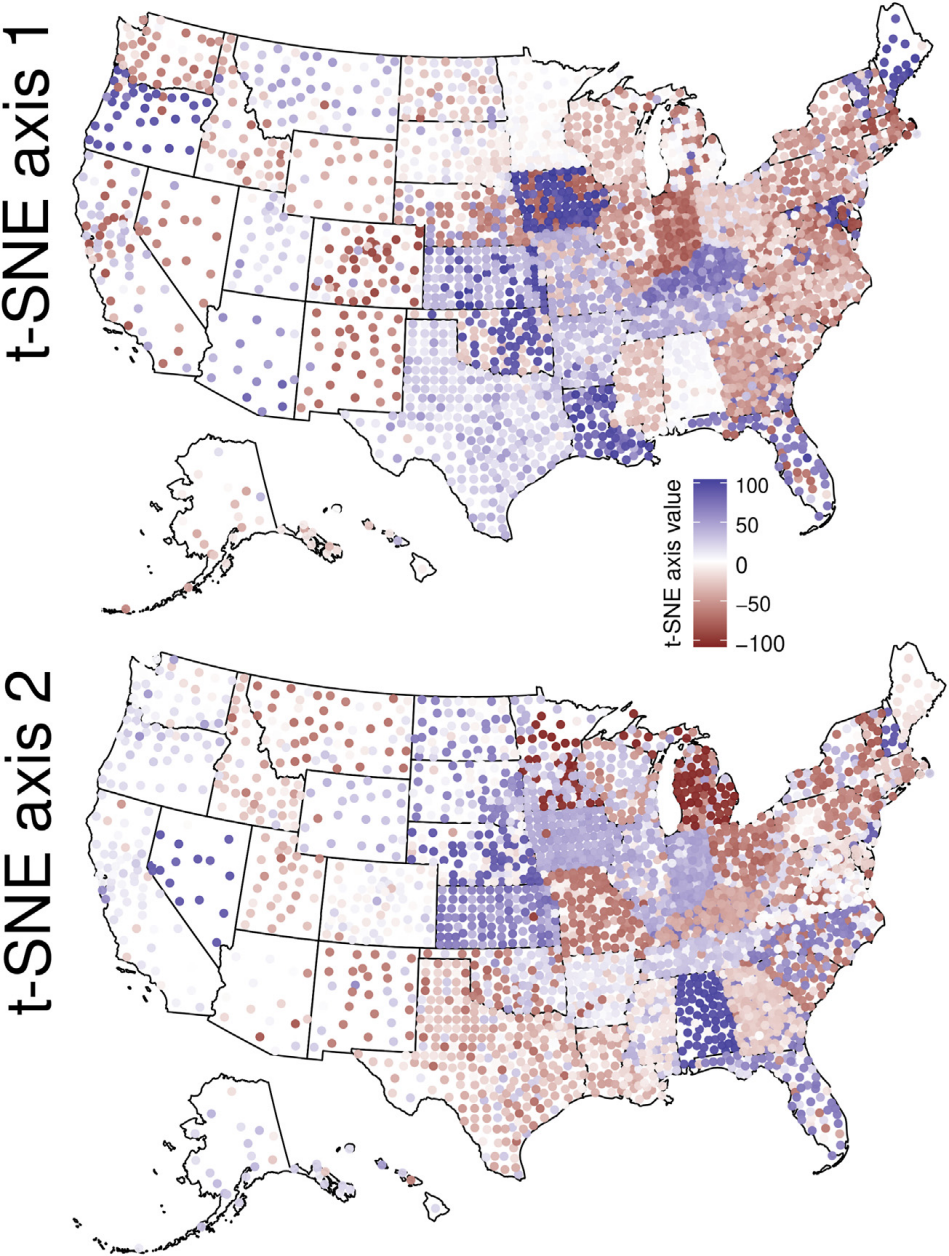
The spatial distribution of epidemic trajectory similarity (t-SNE decomposition of the pairwise dynamic time warping matrix). In this geographic projection of the t-SNE values, there are clearly some states which cluster, suggesting similar mitigation efforts, sampling/reporting biases, and/or epidemic trajectories.

**Fig. 3:**
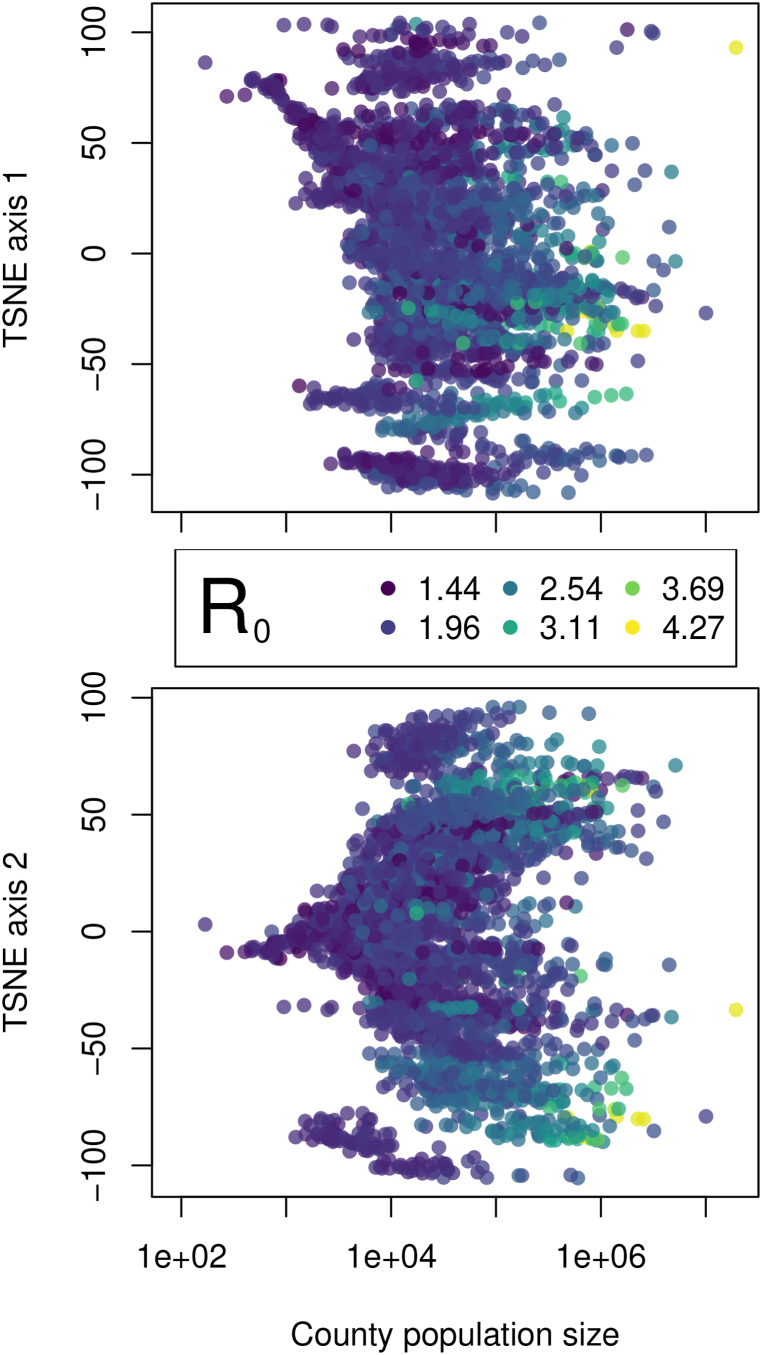
The relationship between t-SNE axes and county population size, with point color corresponding to *R*_0_, highlighting the distribution of t-SNE values, the messy relationship between epidemic similarity and county population size, and the clear scaling of *R*_0_ with county population size.

Pairwise epidemic time series similarity was calculated using dynamic time warping (DTW). These pairwise estimates of similarity were weakly related to Euclidean distance in epidemic time series, suggesting that the DTW approach was able to capture additional information relative to a more simple distance measure (see Supplemental Materials). The matrix of pairwise DTW values were reduced to two axes using t-SNE (Gisbrecht et al., 2015). This low-dimensional representation of site-level epidemic similarity showed clear spatial patterns for the first two t-SNE axes (Fig. 2). Interestingly, the spatial patterns adhere to geopolitical (i.e., US state) boundaries in some instances, a phenomenon which may be due to differences between states in case reporting standards and practices (Sen-Crowe et al., 2021), but is worthy of future investigation. The extent to which geographic distance is related to epidemic similarity is difficult to discern, as we observed spatial structure in population age structure differences (Fig. S3), as well as clear relationships between *R*_0_ and population size (Fig. 3).

### 3.1 What is related to epidemic dissimilarity?

Despite these difficulties, we find a clear relationship between epidemic similarity and geographic distance, age structure dissimilarity, and differences in population size and *R*_0_ between counties (Table 1). These relationships were estimated using Moran's *I*, relating the two axes of epidemic similarity to pairwise matrices describing differences in age structure, geographic distance, *R*_0_, and population size. Moran's *I* is scaled between −1 and 1, where a value of 0 represents a lack of distance-based (or dissimilarity-based) autocorrelation (either negative or positive). All estimated Moran's *I* values in the current analysis were positive, suggesting positive spatial autocorrelation for all dissimilarity and distance matrices examined here. Both t-SNE axes – representing epidemic dissimilarity – were positively related to 1) geographic distance between US counties, 2) age structure dissimilarity, 3) absolute difference in population size, and 4) absolute difference in *R*_0_ (Table 1). Geographic distance was more related to both t-SNE axes relative to age structure, population size, and *R*_0_ based on both the raw observed value and the corresponding standardized *z*-score (Table 1). Differences in *R*_0_ between counties showed the next strongest signal in the t-SNE axes, followed by age structure dissimilarity (Table 1).

**Table 1:** Moran's *I* analysis exploring how t-SNE axes are related to geographic distance, age structure dissimilarity, difference in population size, and difference in *R*_0_. Mantel tests use a randomization approach to generate null distributions to compare observed (obs) to null (exp and sd) distributions. *Z*-scores estimate the divergence of the test statistic from the null distribution.

covariate	t-SNE axis	obs	exp	sd	*p*-value	*z*-score
Geography	1	0.02963	−0.00032	0.00014	<**0.0001**	216.3
	2	0.01930	−0.00032	0.00014	<**0.0001**	141.7
age structure	1	0.00043	−0.00032	0.00001	<**0.0001**	60.5
	2	0.00017	−0.00032	0.00001	<**0.0001**	39.4
population size	1	0.00002	−0.00032	0.00003	<**0.0001**	11.7
	2	0.00004	−0.00032	0.00003	<**0.0001**	12.3
*R*_0_	1	0.00339	−0.00032	0.00003	<**0.0001**	110.7
	2	0.00135	−0.00032	0.00003	<**0.0001**	49.8

## 4 Discussion

Here, we explored how geographic space, demographics, and *R*_0_ influence differences in epidemic trajectories for over 3000 United States counties. We expected – and found – that counties with similar *R*_0_ values tended to have similar epidemics. Independent of this, we found clear effects of geographic distance between counties and dissimilarities in county age structure on resulting epidemic trajectories, suggesting that *R*_0_ estimated from case or mortality data (Ives & Bozzuto, 2021) may not capture the full potential of the epidemic in a given location. Together, we highlight the importance of considering population demographics, age-specific contact network structure, and geographic distance when attempting to estimate epidemic trajectories. While we approach the problem as one of pairwise dissimilarity in epidemics, it may be possible to use similar approaches to recreate an expected epidemic time series for an unsampled location given information on geography and demography.

Spatial structure in both age structure and population sizes precludes the attribution of any form of causal link between age structure or geographic distance and resulting epidemic trajectories. However, our findings, based on the entire epidemic time, broadly agree with similar studies which focused on components of the transmission process or summary statistics such as *R*_0_. Further, the analyses can be updated as the epidemic progresses, or using different time windows to explore how time series clustering changes temporally. It is recognized that both parts of the transmission process – encounter and susceptibility – vary with individual age (Covid et al., 2020; Jones et al., 2021; Kerr et al., 2021; Magpantay et al., 2019), suggesting that for some pathogens including SARS-CoV-2, considering the age structure is quite important to epidemic forecasting (Kerr et al., 2021). Additionally, geographic patterns in *R*_0_ (Ives & Bozzuto, 2021), non-pharmaceutical interventions initiation and compliance (Amuedo-Dorantes et al., 2021; Yang et al., 2021), and vaccine hesitancy (Zuzek et al., 2022) have emerged as potential drivers for spatial variation in epidemic progression (Richards et al., 2022). By comparing epidemic trajectories directly, using a flexible framework which allows epidemics to be sampled at different timescales, we have found that these similarity patterns in summary values, transmission components, and intervention uptake scale up directly to the similarity between entire epidemics.

One major result is the marked state-level clustering of epidemic similarity (Fig. 2). Previous clustering of US states was observed early in the pandemic at the state-level (Rojas et al., 2020), potentially reflecting large scale differences in mitigation protocols (e.g., closing bars and restaurants) or differences in testing regimes across US states. The consistent clustering at US state level when considering counties as the unit of study suggests that state-level variation in reporting, testing, or mitigation may manifest to influence epidemic similarity. Understanding the cause of this clustering may help to inform mitigation efforts, and help to uncover differences in testing or reporting that may be important to understand spatial patterns of infectious disease.

It is interesting that epidemic similarity showed clear signals of geographic distance, age structure, and county-level differences in population size and *R*_0_, given that counties also varied in other marked ways. For instance, differences in non-pharmaceutical interventions, vaccination rate variation, and other demographic factors which we recognize are important to pathogen spread (Abedi et al., 2021; Ge et al., 2022; Zuzek et al., 2022) did not mask the effect of age structure. One reason for this may be that age structure is serving as a surrogate for other measures of population demography not inherently related to age-structured transmission. That is, differences in vaccination hesitancy (Zuzek et al., 2022) and risk perception (Bruine de Bruin, 2021) may differ across age groups. One way to parse this out would be to examine epidemic trajectory similarity in other geopolitical locations and at different spatial scales, where the relative influence of geographic connectivity, population demographics, and pathogen strain diversity may be quite different. The incorporation of temporal information on mitigation efforts, strain diversity, and availability of health care infrastructure is a clear next step to understanding and forecasting epidemic time series. This effort is obviously not aimed at forecasting directly, but could potentially be used to infer approximate epidemic dynamics for future epidemics or to explore how deviations from epidemic trajectories between neighboring counties (or those with similar age structure) may be driven by other critical variables.

The COVID-19 pandemic will not be the last pandemic (Medicine, 2022), and understanding the factors which influence epidemic dynamics are intrinsically important to public health measures. Perhaps this current pandemic is a special case, as comparisons in *R*_0_ between SARS-CoV2 and 1918 pandemic influenza revealed little consensus in heavily impacted cities (Foster et al., 2022). But it seems relevant to use approaches such as the one we do here to understand how epidemic trajectories differ, both within the same pandemic and potentially for different pathogens (e.g., how dissimilar are temporal patterns in seasonal flu epidemics in a given location?). The comparison of epidemic trajectories – especially along moving windows as the epidemic progresses – can provide insight into the relative effects of different mitigation and control efforts. Finally, while many approaches to forecasting epidemics rely on a single time series, this work alludes to the possibility of incorporating information on nearby or similar time series, creating the possibility of joint epidemic forecasts.

## Funding source

This work has been supported by the U.S. National Science Foundation RAPID grant (NSF-DEB-2031196).

## Ethical approval statement

The present study used publicly available data compiled by the Center for Systems Science and Engineering at Johns Hopkins University (Dong et al., 2020).

## Author contributions

TAD performed the analysis. All authors contributed to manuscript writing.

## Data accessibility

*R* code is available on figshare at

https://doi.org/10.6084/m9.figshare.19782406.v1 .

## Declaration of competing interest

The authors have no conflicts of interest to declare.
