# Supplementary material for: Epidemic time series similarity is related to geographic distance and age structure
Source: Infect Dis Model. 2022 Oct 12;7(4):690–7. doi: 10.1016/j.idm.2022.09.002 (PMC9579807; doi:10.1016/j.idm.2022.09.002)
Supplement: Multimedia component 1 [file mmc1.pdf]

Supplementary materials

Title: Epidemic time series similarity is related to geographic distance and age structure

Authors: Tad A Dallas, Grant Foster, Robert Richards, & Bret D Elderd

Does time need to warped?

We use dynamic time warping as a flexible way to compare time series similarity. Here, we explore how much of this signal would be observed if we simply calculated the summed difference in pairwise epidemic trajectories. We found the two approaches are roughly similar, but that the dynamic time warping does result in different estimates of epidemic similarity (Figure S1), highlighting the application of such time series approaches to epidemic trajectory data.

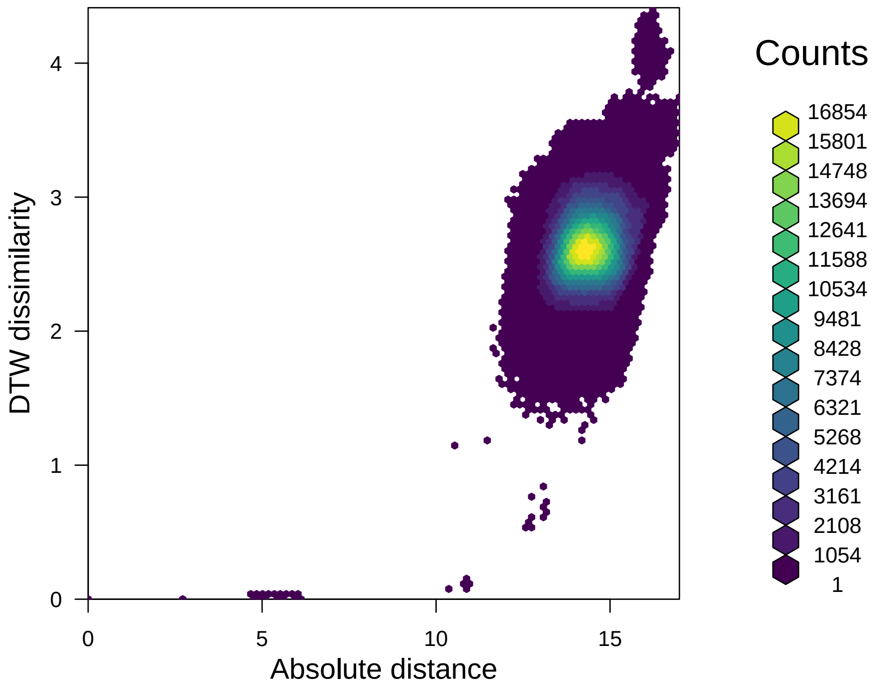

Figure S1: The sum of the absolute difference between the two time series is related to the dynamic time warp dissimilarity in this particular application. There are still clear differences between the two.

Truncating the epidemic time series

In the main text, we considered the full epidemic time series, including case counts in which case counts were zero-valued. Here, we explore to what extent this influences the dynamic time warping estimates, and our overall results. This does not influence our overall results (Table S1), and the two estimates of epidemic dissimilarity produced by truncating the epidemic time series versus keeping the entire time series are quite positively related (Figure S2).

Table S1: Moran's I analysis exploring how t-SNE axes are related to geographic distance, age structure dissimilarity, difference in population size, and difference in R_0. Mantel tests use a randomization approach to generate null distributions to compare observed (`obs`) to null (`exp` and `sd`) distributions. Z-scores estimate the divergence of the test statistic from the null distribution.

covariate	t-SNE axis	obs	exp	sd	p-value	z-score
geography	1	0.01832	-0.00032	0.00014	$<$ **0.0001**	134.7
	2	0.02849	-0.00032	0.00014	$<$ **0.0001**	208.1
age structure	1	0.00041	-0.00032	0.00001	$<$ **0.0001**	58.9
	2	0.00024	-0.00032	0.00001	$<$ **0.0001**	45.4
population size	1	0.00001	-0.00032	0.00003	$<$ **0.0001**	11.2
	2	0.00008	-0.00032	0.00003	$<$ **0.0001**	13.7
R_0	1	0.00231	-0.00032	0.00003	$<$ **0.0001**	78.6
	2	0.00106	-0.00032	0.00003	$<$ **0.0001**	41.2

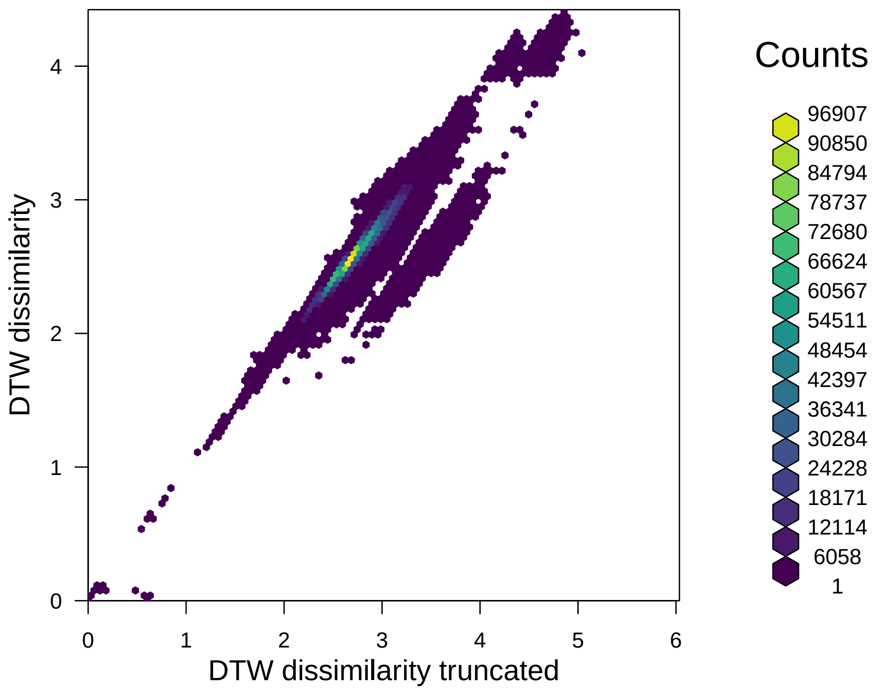

Figure S2: The relationship between dynamic time warping estimates when the time series was truncated to remove the majority of zero values (x-axis) compared to when the entire epidemic time series was used (y-axis). Small variations do exist, but this does not affect our overall findings.

R_0, population size, and epidemic similarity

While we can consider epidemics in US counties as being quasi-isolated, with travel restrictions and differing epidemic timing, it is not possible to control for the inherent link between R_0 (which is estimated from epidemic time series themselves) and population size (Figure S4) and the resulting epidemic trajectory similarity values obtained from the t-SNE decomposition of the pairwise dynamic time warping matrix of epidemic similarity.

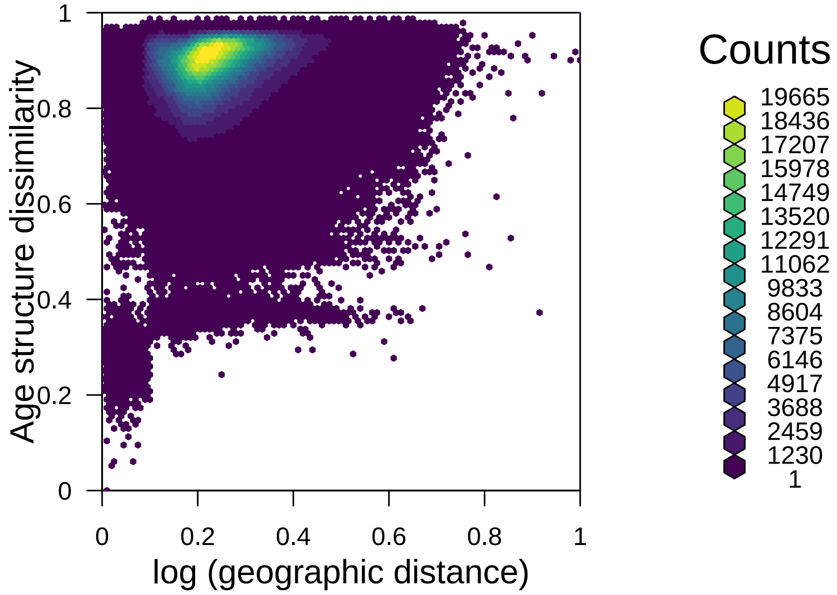

Figure S3: The relationship between geographic distance and age structure dissimilarity.

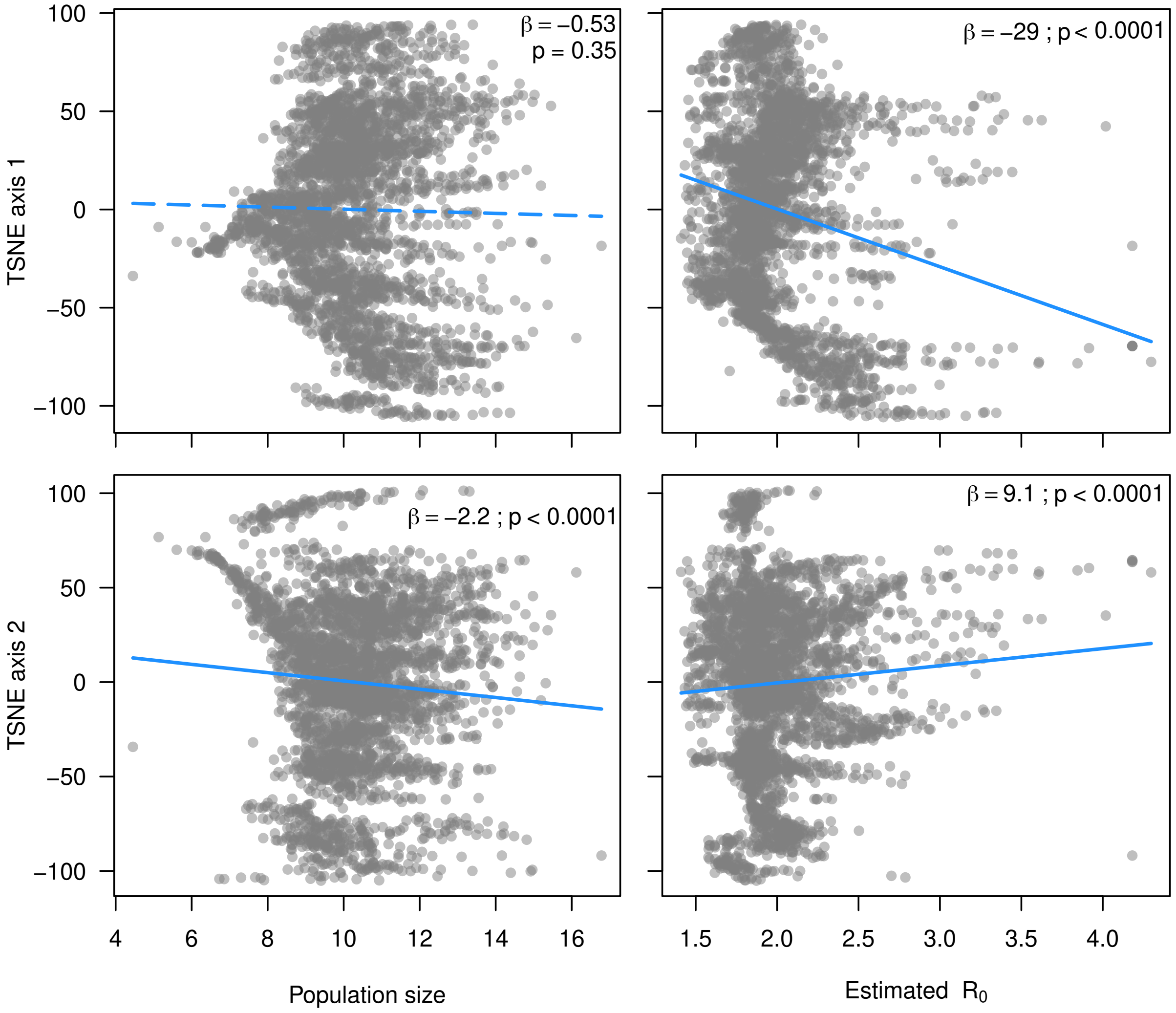

Figure S4: The relationship between epidemic dissimilarity (t-SNE axes as y-axes) and popularion size (first column) and estimated R_0 (second column). Blue lines are linear fits (with associated β and p-values in each panel), where significant lines are solid.

Epidemic similarity as a function of geopolitical boundaries

Epidemic similarity, when compressed to the two t-SNE axes, showed clear US state-level relationships. There are numerous potential reasons for this, including state-level implementations of lockdown orders, variation in state-level testing efforts, and variability in reporting. These are beyond the scope of the current work, but it seems prudent to highlight this variation in t-SNE space (Figure S5).

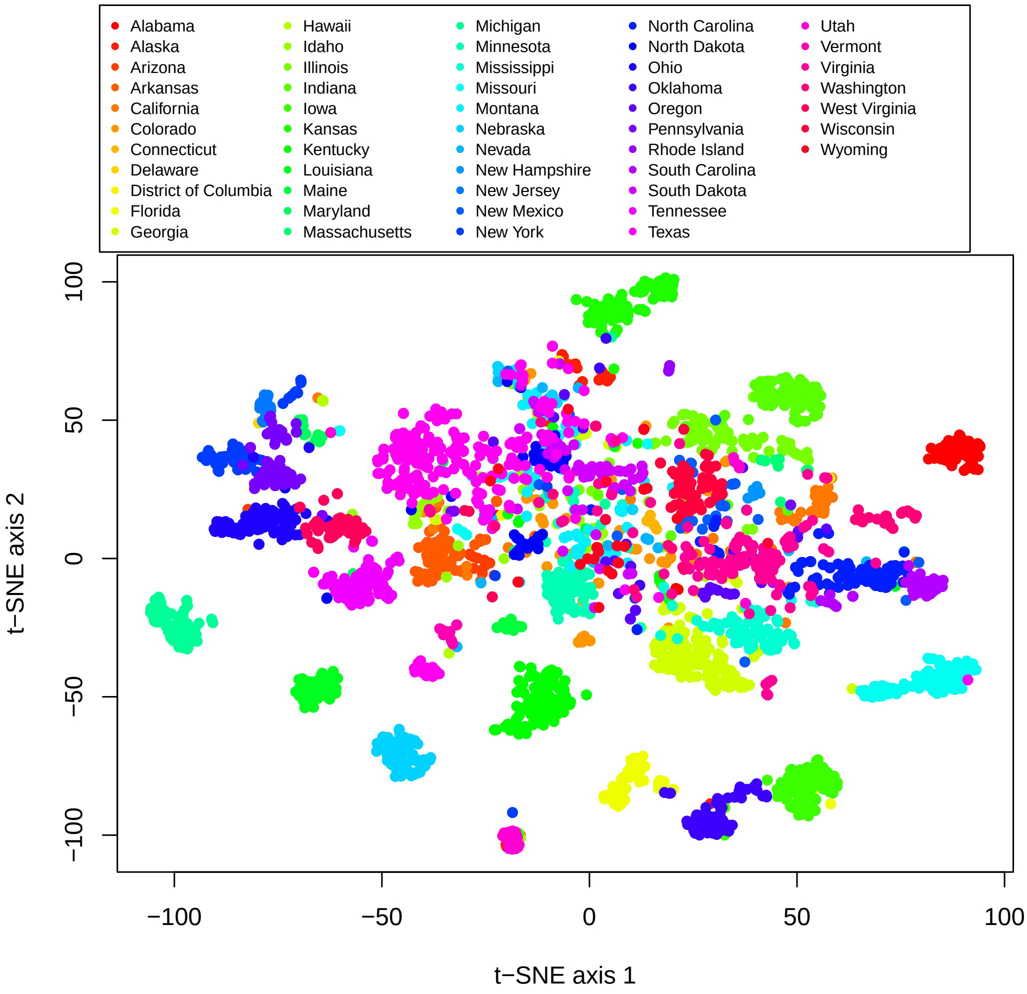

Figure S5: Epidemic similarity in t-SNE space shows clear state-level clustering, suggesting that epidemic similarity was related to some aspect of this geopolitical scale, such as variable mitigation, testing, and reporting efforts.

Mantel Tests

Here, we explore how the pairwise epidemic similarity is related to the distance (or dissimilarity) matrices related to demography and spatial processes. If we claim that z-score as a measure of association between epidemic trajectory similarity and geographic distance, age structure dissimilarity, population size difference, and R_0 difference, then we would conclude that geographic distance and R_0 difference between US counties are the *most* related to epidemic similarity. Each of the distance or dissimilarity matrices were significantly related to the pairwise epidemic dissimilarity matrix. Taking the estimated z-score from the Mantel tests as a measure of association would lead us to conclude that geographic distance was far less important than other matrices. Considering the inherent collinearity between many of these variables, the most salient aspect of this becomes that all of these demographic and spatial factors were significantly related to epidemic similarity.

Table S2: Mantel tests – permutation tests relating two pair-wise dissimilarities to one another – found that geographic distance, age structure dissimilarity, difference in population size, and difference in R_0 were all related to epidemic trajectory dissimilarity.

covariate	z	p
geography	3111220	$<$ **0.001**
age structure	11354792	$<$ **0.001**
population size	11218853	$<$ **0.001**
R_0	12819318	$<$ **0.001**
